# Toxin B Variants from *Clostridium difficile* Strains VPI 10463 and NAP1/027 Share Similar Substrate Profile and Cellular Intoxication Kinetics but Use Different Host Cell Entry Factors

**DOI:** 10.3390/toxins11060348

**Published:** 2019-06-17

**Authors:** Diana López-Ureña, Josué Orozco-Aguilar, Yendry Chaves-Madrigal, Andrea Ramírez-Mata, Amanda Villalobos-Jimenez, Stefan Ost, Carlos Quesada-Gómez, César Rodríguez, Panagiotis Papatheodorou, Esteban Chaves-Olarte

**Affiliations:** 1 Facultad de Microbiología and Centro de Investigación en Enfermedades Tropicales, Universidad de Costa Rica, 10101 San José, Costa Rica; diana.lopezurena@ucr.ac.cr (D.L.-U.); yencu91@gmail.com (Y.C.-M.); andre.rm28@gmail.com (A.R.-M.); amandalucia94@gmail.com (A.V.-J.); carlos.quesada@ucr.ac.cr (C.Q.-G.); cesar.rodriguezsanchez@ucr.ac.cr (C.R.); 2 Facultad de Farmacia and Laboratorio de Ensayos Biológicos, Escuela de Medicina, Universidad de Costa Rica, 10101 San José, Costa Rica; josue.orozco@ucr.ac.cr; 3 Institut für Experimentelle und Klinische Pharmakologie und Toxikologie, Albert-Ludwigs-Universität Freiburg, D-79104 Freiburg, Germany; stefan.ost@gmx.net; 4 Institut für Pharmakologie und Toxikologie, Universitätsklinikum Ulm, D-89081 Ulm, Germany; panagiotis.papatheodorou@uni-ulm.de

**Keywords:** *Clostridium difficile*, NAP1/027 toxin B, receptor binding, frizzled receptors

## Abstract

*Clostridium difficile* induces antibiotic-associated diarrhea due to the release of toxin A (TcdA) and toxin B (TcdB), the latter being its main virulence factor. The epidemic strain NAP1/027 has an increased virulence attributed to different factors. We compared cellular intoxication by TcdB_NAP1_ with that by the reference strain VPI 10463 (TcdB_VPI_). In a mouse ligated intestinal loop model, TcdB_NAP1_ induced higher neutrophil recruitment, cytokine release, and epithelial damage than TcdB_VPI_. Both toxins modified the same panel of small GTPases and exhibited similar in vitro autoprocessing kinetics. On the basis of sequence variations in the frizzled-binding domain (FBD), we reasoned that TcdB_VPI_ and TcdB_NAP1_ might have different receptor specificities. To test this possibility, we used a TcdB from a NAP1 variant strain (TcdB_NAP1v_) unable to glucosylate RhoA but with the same receptor-binding domains as TcdB_NAP1_. Cells were preincubated with TcdB_NAP1v_ to block cellular receptors, prior to intoxication with either TcdB_VPI_ or TcdB_NAP1_. Preincubation with TcdB_NAP1v_ blocked RhoA glucosylation by TcdB_NAP1_ but not by TcdB_VPI_, indicating that the toxins use different host factors for cell entry. This crucial difference might explain the increased biological activity of TcdB_NAP1_ in the intestine, representing a contributing factor for the increased virulence of the NAP1/027 strain.

## 1. Introduction

*Clostridium difficile* is a relevant nosocomial pathogen because of its role in antibiotic-associated diarrhea and pseudomembranous colitis. These infections usually occur in hospitalized patients that receive antibiotic treatment. Under these conditions the microbiota is altered, allowing the germination and colonization by *C. difficile*. Upon proliferation, this bacterium releases two toxins responsible for all the signs and symptoms of the disease, i.e., toxin A (TcdA) and toxin B (TcdB) [1]. Despite some controversy, it has been proposed that TcdB is the main virulence factor of *C. difficile* [2,3]. These two toxins are multi-domain proteins of high molecular weight from the family of Clostridial Glucosylating Toxin (CGTs) and follow a multi-step strategy to intoxicate eukaryotic cells [4]. As for TcdB, it initially binds to receptors located on the cell surface. Three different receptors have been identified for TcdB of VPI 10463, i.e., the poliovirus receptor-like 3 (PVRL3), chondroitin sulfate proteoglycan 4 (CSPG4), and certain Wnt receptors of the frizzled family (namely, FZD1, FZD2, and FZD7) [5,6,7]. Once the toxin binds to these receptors, it enters the cell by receptor-mediated and clathrin-dependent endocytosis in a compartment that undergoes acidification [8]. This condition triggers a conformational change in the protein, allowing the insertion of hydrophobic domains and the translocation of the N-terminal end to the cytosol. Exposure to inositol hexakisphosphate (InsP6) located in the cytoplasm activates an autoprocessing activity that cleaves the protein and releases the enzymatic domain, which in turn glucosylates, and thus inactivates, members of the family of small GTPases [1,9]. This sequential and concerted action leads to the collapse of the actin cytoskeleton and to a generalized disruption of signaling processes mediated by small GTPases of the Rho subfamily. In the intestine, this cytotoxicity process is manifested by the disruption of the epithelial monolayer and the proinflammatory activation of both epithelial and immune cells [1,10,11].

*C. difficile* is an extremely variable species, with a core genome reported to be as low as 30% [12,13]. This diversity is reflected in the emergence of strains with varying degrees of virulence, such as the epidemic strain NAP1/027. This strain has been responsible of numerous outbreaks worldwide [14,15,16,17]**,** and its increased virulence has been attributed to a plethora of phenotypic and genotypic characteristics, including increased toxin secretion, a mutation in the PaLoc negative regulator *tcdC*, production of a binary toxin, increased sporulation, and production of a TcdB with increased biological activity [18,19,20,21].

In this report, we compared TcdB from the reference strain VPI 10463 (TcdB_VPI_) with the corresponding toxin purified from a NAP1/027 strain (TcdB_NAP1_) at different steps of the intoxication process. In addition, by using a variant NAP1 TcdB previously reported to differ only in the glucosyltransferase domain (TcdB_NAP1v_) [22], we found evidence for differences in the exploitation of host cell entry factors between TcdB_VPI_ and TcdB_NAP1_. Thus, we postulate the existence of different or additional receptors for TcdB_NAP1_, providing a possible explanation for its increased biological activity.

## 2. Results

### 2.1. TcdB_NAP1_ Has an Increased Biological Activity in the Intestine

**Figure 1:**
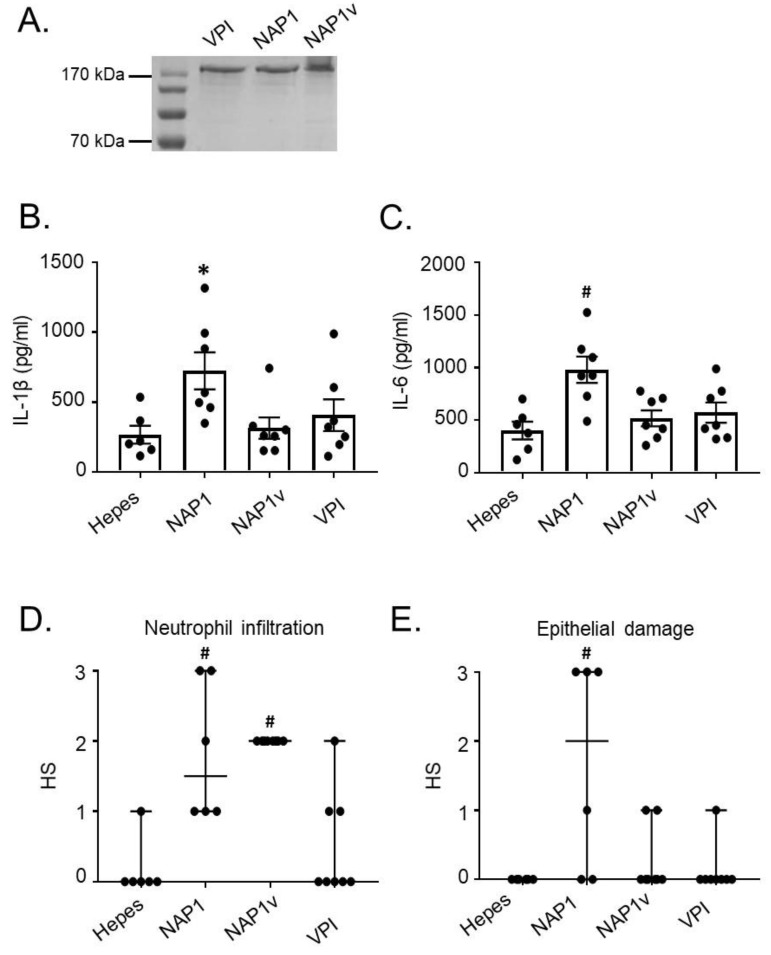
Toxin B (TcdB) from NAP1 induces a higher inflammatory influx and ileal disruption. The in vivo effect of various TcdBs was assayed in a murine ligated ileal loop model. (**A**) TcdBs were obtained from the supernatants of strains grown in a dialysis system culture and purified by ion-exchange chromatography and gel filtration. The purity of the toxins was assessed after SDS-PAGE by Coomassie staining of a 7.5% gel loaded with 1.5 µg of TcdB_VPI_ (VPI), TcdB_NAP1_ (NAP1), and TcdB_NAP1v_ (NAP1v). Then, ileal loops were treated for 4 h with 10 μg of purified TcdB_VPI_ (VPI), TcdB_NAP1_ (NAP1), and TcdB_NAP1v_ (NAP1v); (**B**,**C**) The effect of the toxins on inflammatory cytokines was measured; (**D**,**E**) Neutrophil infiltration and epithelial damage induced by the toxins was also determined using a histopathological score (HS) scale from 1 (mild) to 3 (severe). Hepes was used as a negative control. Error bars represent means ± SEM (**A**,**B**) and median ± range (**C**,**D**), *n* ≥ 5; * *p* < 0.05 compared to Hepes, # *p* < 0.05 compared to other groups (One-way ANOVA with Bonferroni’s correction, Kruskal–Wallis test, and Dunn’s multiple-comparison test).

A previous report indicated that TcdB_NAP1_ possesses an increased biological activity compared to reference TcdB in the zebra fish model [19]. We tested whether the same was true in a mice model. Purified toxins from the hyper-producing strains NAP1, NAP1v, and VPI 10463 were used for all the experiments (Figure 1). Ligated intestinal loops were inoculated with 10 μg of TcdB_NAP1,_ TcdB_VPI_, or TcdB_NAP1v_ and after 4 h the proinflammatory response was analyzed measuring the levels of ileal cytokines. TcdB_NAP1_ induced a significantly higher release in the intestinal mucosa of IL-1β and IL-6 than TcdB_VPI_ and TcdB_NAP1v_ (Figure 1). This increased activity of TcdB_NAP1_ was also seen when epithelial damage was evaluated by histology (Figure 1). In the case of neutrophil migration, both TcdB_NAP1_ and TcdB_NAP1v_ showed an increased activity when compared to TcdB_VPI_ (Figure 1).

### 2.2. The Cytotoxic Potency of TcdB_NAP1_ Depends on the Cell Line Evaluated

**Figure 2:**
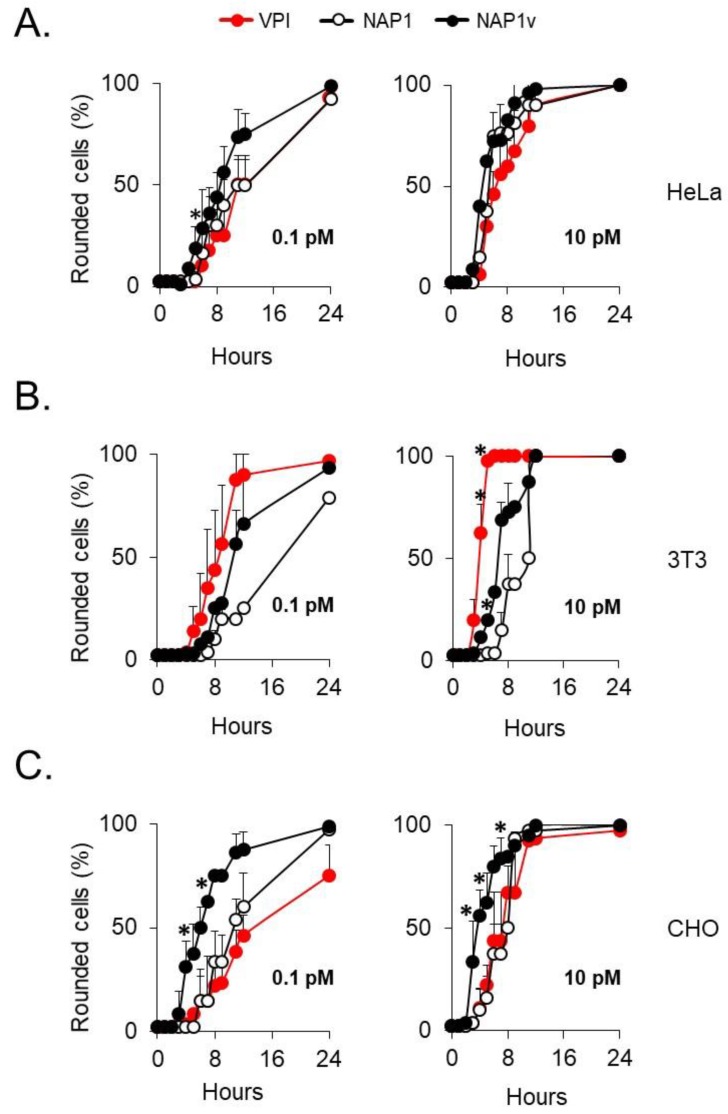
Cell rounding of distinct cell lines induced by TcdBs. (**A**) HeLa, (**B**) 3T3 fibroblasts, and (**C**) CHO cells were treated with the indicated concentrations of TcdB_VPI_ (VPI), TcdB_NAP1_ (NAP1), and TcdB_NAP1v_ (NAP1v). The percentage of round cells in each well was evaluated every hour for a period of 12 h and then after 24 h from the start of the experiment. Error bars represent means ± SD of 100 cells in three independent experiments; * *p* < 0.05 (One-way ANOVA with Bonferroni´s correction).

To determine whether the increased biological activity induced by TcdB_NAP1_ was due to an increased cytotoxic activity, we titrated the toxins in three cell lines. In HeLa cells, the three toxins displayed a similar cytopathic titer (Figure 2A). In contrast, TcdB_VPI_ had a significantly higher cytopathic titer than TcdB_NAP1_ and TcdB_NAP1v_ in 3T3 fibroblasts (Figure 2B). Interestingly, the opposite was found in CHO cells, in which both TcdB_NAP1_ and TcdB_NAP1v_ presented a higher cytopathic titer than TcdB_VPI_ (Figure 2C). These results demonstrate that the different cytotoxic potencies depend on the target cell line rather than on an intrinsic characteristic of the toxins.

**Figure 3:**
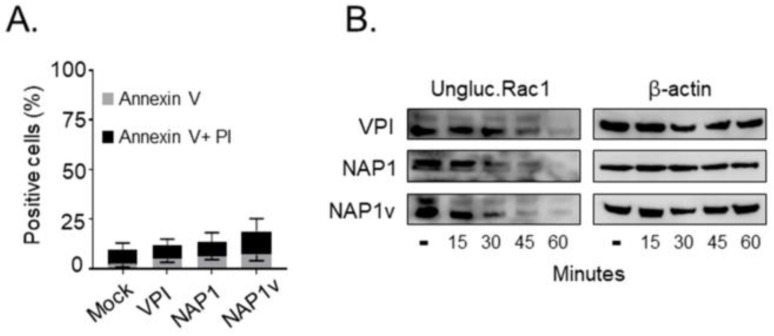
Cytotoxicity of TcdBs in HeLa cells. (**A**) HeLa cells were treated with 100 pM of TcdB_VPI_ (VPI), TcdB_NAP1_ (NAP1), and TcdB_NAP1v_ (NAP1v) for 24 h. Cytotoxicity was analyzed by flow cytometry using propidium iodide (PI)/anexin V double staining. Control cells were left untreated (mock). Error bars represent means ± SD of three independent experiments; (**B**) Following the addition of 100 pM of TcdB, the cells were lysed at the indicated time points, and the dynamics of Rac1 glucosylation was monitored by immunoblot with a specific anti-Rac1 antibody that only recognizes the unmodified form of this protein (ungluc.Rac1). Untreated cells (-) were included as a positive control for unglucosylated Rac1, and immunodetection of beta-actin served as a loading control (β-actin). Shown are representative western blot images from three independent experiments.

We next evaluated the ability of the three toxins to induce cell death using propidium iodide (PI)/anexin V double staining, which evaluates early apoptotic events (anexin V-positive) and late apoptotic or necrotic cell death (anexin V- and PI-positive). HeLa cells intoxicated with either of the three toxins for 24 h had a similar response, displaying a low percentage of cytotoxicity (below 20%) (Figure 3A). At the toxin concentrations used, the cells already showed a visible cytopathic effect after 2 h of treatment (data not shown), indicating that none of the toxins had an increased ability to induce cell death even if they had fully modified their intracellular substrates with a similar dynamic, as shown by the glucosylation of Rac1 (Figure 3B).

### 2.3. TcdB_NAP1_ and TcdB_VPI_ Possess Similar Enzymatic Activities

**Figure 4:**
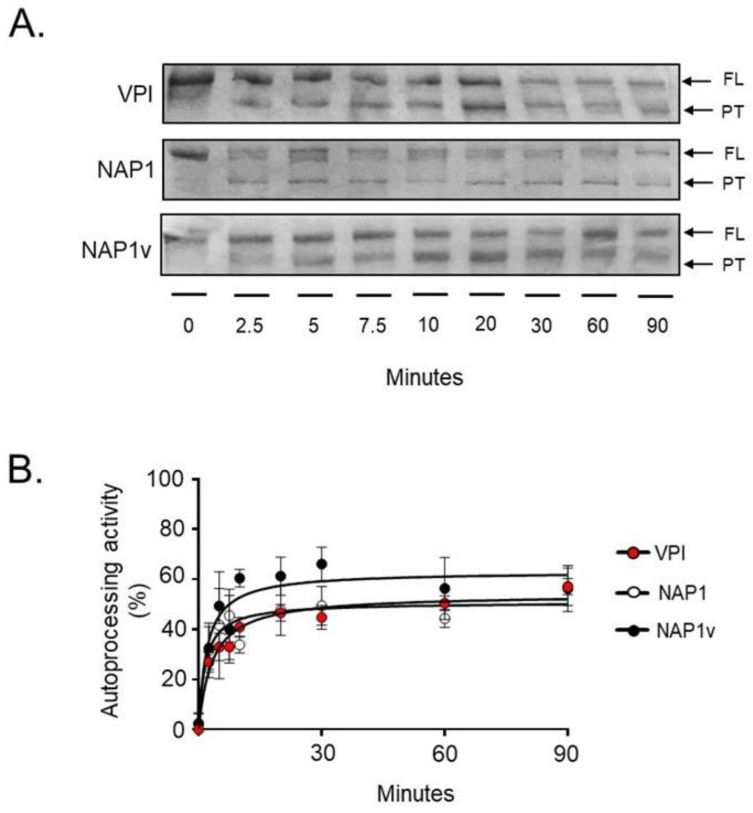
In vitro autoprocessing of TcdB. The in vitro processing of TcdB_VPI_ (VPI), TcdB_NAP1_ (NAP1), and TcdB_NAP1v_ (NAP1v) was evaluated in the presence of 10 mM of the co-factor inositol hexakisphosphate (InsP6). The reactions were incubated at 37°C for the indicated times, and toxins or toxin fragments were then detected by Coomassie Blue staining. The enzymatic activity was determined by the presence of full-length (FL) toxin (1–2366), which decreased over time, and processed toxin (544–2366, PT), which increased over time. Shown in (**A**) is one representative SDS-PAGE gel from three independent experiments; (**B**) The bands corresponding to the FL toxin and the PT were quantified by densitometry. The autoprocessing activity of each toxin was determined by comparing the relative amounts of both forms of the toxin, and the percentage obtained was plotted using nonlinear regression. Error bars represent means ± SD of three independent experiments.

Two enzymatic activities are crucial for the intoxication of cells by TcdB: (1) the autoprocessing activity elicited by InsP6 and (2) the glucosyltransferase activity on small GTPases [4,9,23]. We compared both activities to determine whether differences at this level might explain the increased biological activity of TcdB_NAP1_. To this end, purified toxins were exposed in vitro to InsP6, and autoproteolysis was followed by detection of a proteolytic product corresponding to residues 544–2366 [24]. Under these conditions, the autoproteolytic event was initiated after only 2.5 min and proceeded efficiently in the three toxins (Figure 4A). Quantitation of the process did not reveal statistical differences among the autoprotolytic activities of the three toxins (Figure 4B).

**Figure 5:**
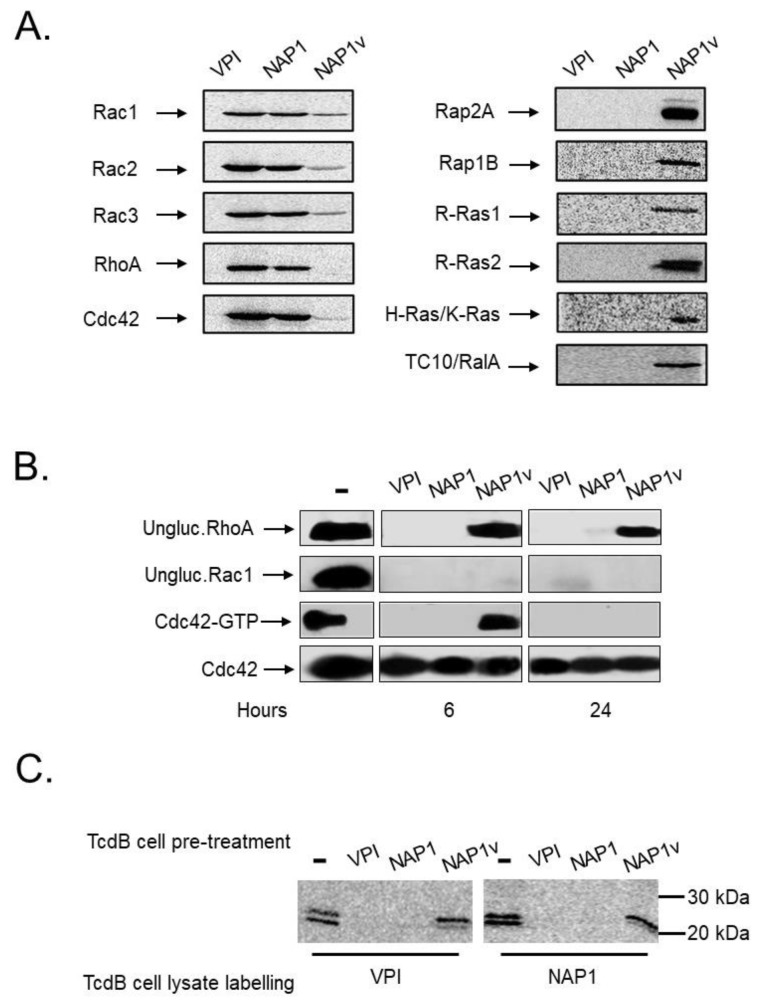
Glucosyltransferase activity of TcdBs. (**A**) The in vitro glucosylation of distinct recombinant GTPases by TcdB_VPI_ (VPI), TcdB_NAP1_ (NAP1), and TcdB_NAP1v_ (NAP1v) was determined using UDP-[14C] glucose as a co-substrate. The reactions were incubated for 1 h and then subjected to SDS-PAGE. The radiolabeled bands were detected by phosphor imaging; (**B**) Following the addition of 100 pM of TcdB, HeLa cells were lysed at the indicated times, and the glucosylation of Rac1 and RhoA was monitored by immunoblot with specific anti-Rac1 and anti RhoA antibodies that only recognize the unmodified form of the proteins (ungluc.Rac1 and ungluc.RhoA, respectively). The effect of TcdBs on the activation state of Cdc42 was also evaluated. After intoxication, one part of the lysate was used as a control for the total amount of GTPase, and the rest was incubated with Rho Binding Domain–GST-sepharose beads. Cdc42 was detected by immunoblot with anti-Cdc42 antibodies; (**C**) HeLa cells were treated with 10 pM of TcdB_VPI_ (VPI), TcdB_NAP1_ (NAP1), and TcdB_NAP1v_ (NAP1v) for 12 h (TcdB cell pre-treatment). The cells were lysed, and the lysate proteins were glucosylated in vitro by TcdB_VPI_ (VPI) or TcdB_NAP1_ (NAP1), according to the conditions stated in (**A**) (TcdB cell lysate labelling). Untreated cells (-) in (**B**) and (**C**) were included as a positive control for unmodified or activated proteins.

The substrates modified by the three toxins were determined in vitro using recombinant small GTPases and UDP-[14C]glucose. Both TcdB_NAP1_ and TcdB_VPI_ glucosylated an identical panel of substrates, with preference for Rho GTPases (Rho, Rac, and Cdc42) (Figure 5). On the other hand, TcdB_NAP1v_ glucosylated Ras GTPases (Rap, Ras, and Ral) and to a lower degree, Rac and Cdc42 isoforms (36% and 45% TcdB_NAP1v_-induced Rac1 glucosylation compared to glucosylation by TcdB_VPI_ and TcdB_NAP1_, respectively; 23% and 25% TcdB_NAP1v_-induced Cdc42 glucosylation compared to glucosylation by TcdB_VPI_ and TcdB_NAP1_ respectively), (Figure 5B), yet, it did not modify RhoA (Figure 5A). These substrate specificities were confirmed ex vivo following the modification of Rho and Rac with the use of glucosylation-sensitive antibodies or Cdc42 in GTPase activation assays (Figure 5B). Since these results suggested that TcdB_NAP1_ and TcdB_VPI_ modify the same intracellular substrates, we applied a differential glucosylation approach to confirm this. Cells were intoxicated with TcdB_NAP1,_ TcdB_NAP1v,_ or TcdB_VPI_ until a full cytopathic effect (CPE) developed. Lysates were then prepared and used as substrates for radioactive in vitro glucosylation. Both TcdB_NAP1_ and TcdB_VPI_ were able to radioactively label substrates in the lysates from TcdB_NAP1v_-intoxicated cells (Figure 5C). However, neither TcdB_NAP1_ nor TcdB_VPI_ were able to label any GTPase in lysates from cells previously intoxicated by themselves, demonstrating unequivocally that both toxins target the same GTPases ex vivo (Figure 5C).

These results altogether indicate that the difference in biological activity between TcdB_NAP1_ and TcdB_VPI_ toxins does not reside in intrinsic enzymatic factors.

### 2.4. TcdB_NAP1_ and TcdB_VPI_ Exploit Different Host Factors for Cell Entry

**Figure 6:**
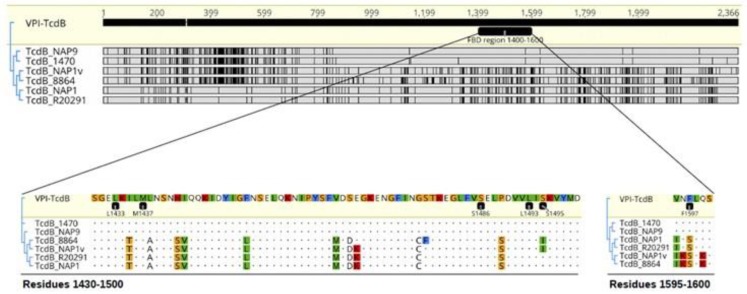
Comparison of TcdB residues involved in frizzled receptor binding. Tree alignment of TcdB sequences of strains VPI 10493, 1470 (reference TcdB variant strain), NAP9 (TcdB variant strain), 8864 (TcdB variant strain), NAP1v, R20291 (NAP1 reference strain), and NAP1. TcdB_VPI_ was used as a reference. Discrepancies are highlighted with black bars or colored-residue letters.

Prompted by the previous results, we decided to focus on the receptor binding properties of these toxins. We searched for differences at the amino acid level in their frizzled binding domain region (FBD) of various TcdBs (Figure 6), which is involved in the palmitoleic acid (PAM)-mediated interaction between the FBD of TcdB_VPI_ and the frizzled receptor through the cysteine-rich domain of this eukaryotic membrane protein (TcdB-FBD–CRD2) [25]. The results obtained were clearly grouped into two distinguishable clusters (Figure 6; upper part), and considerable sequence variations between TcdB_NAP1_ and TcdB_VPI_ were obvious within the entire FBD (Figure 6; upper part). Residues L1433, M1437, S1486, L1493, and S1495, which interact with the PAM tail protruding from the CRD2 groove, were conserved (Figure 6). By contrast, TcdB_NAP1_ and TcdB_VPI_ differed in residue 1597, which, according to a recent 2.5-Å-resolution crystal structure, stabilizes the middle part of PAM and is crucial for PAM binding [25].

**Figure 7:**
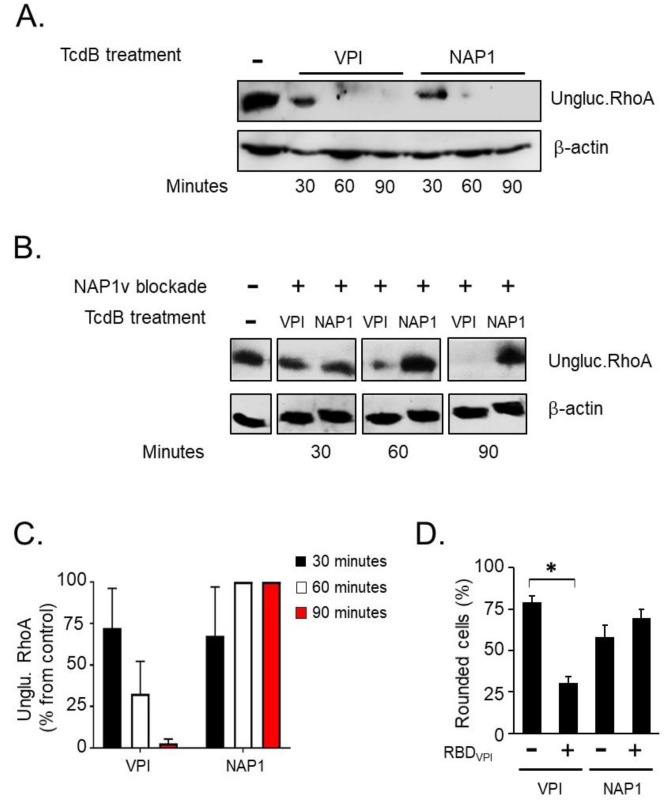
Uptake of TcdB from TcdB_NAP1_ is blocked by TcdB_NAP1v_. In order to determine whether the different toxins use the same host cell entry factors, an uptake competition assay was performed in HeLa cells. (**A**) The cells were treated with 10 pM of TcdB_VPI_ (VPI) or TcdB_NAP1_ (NAP1) for the indicated times at 37 °C. Following cell lysis, the uptake of each toxin was evaluated by western blot using an antibody that only binds to the unglucosylated form of RhoA GTPase (ungluc.RhoA); (**B**) The cells were treated with 1 nM of TcdB_NAP1v_ (NAP1v) for 30 min at 4 °C to induce binding of TcdB_NAP1v_ to the cell but not its uptake (NAP1v blockade +). The cells were then treated with 10 pM of TcdB_VPI_ or TcdB_NAP1_ and incubated for 30, 60, and 90 min at 37 °C (TcdB treatment). Toxin uptake was determined by immunodetection of unmodified RhoA, as stated in (A). Untreated cells (-) were included as a positive control for unglucosylated Rac1, and immunoblot of β-actin served as a loading control (β-actin). Shown are representative western blot images from three independent experiments; (**C**) The bar graph shows the amount of unglucosylated RhoA in intoxicated cells relative to the unglucosylated RhoA amount in untreated cells, which was set to 100%. Error bars represent means ± SD of three independent experiments; (**D**) 10 nM of recombinant Receptor Binding Domain (RBD_VPI_) corresponding to TcdB_1349-1811_ of VPI 10463 was added to the cells, and these were incubated for 30 min at 4 °C to block toxin receptors. Control cells were mocked-treated with the corresponding volume of buffer and also incubated for 30 min at 4 °C (RBD_VPI_ -). The cells were then treated with 100 pM of TcdB_VPI_ or TcdB_NAP1_, and the percentage of round cells was evaluated after 60 min as a parameter of full-length toxin uptake. Error bars represent means ± SD of 1000 cells in triplicate samples; * p < 0.05 (Student *t* test). This experiment is representative of three independent experiments with similar results.

We speculated that TcdB_NAP1_ has a receptor specificity different from that of TcdB_VPI_ following these observations: (i) we did not find intrinsic differences in the enzymatic activities of the two toxins, (ii) the relative cytotoxic potencies of the toxins depended on the cell line tested, and (iii) the toxins present significant differences at the sequence level in the FBD, as explained above (Figure 6). To test our hypothesis, we took advantage of the sequence similarity of the receptor-binding domains of TcdB_NAP1_ and TcdB_NAP1v_ and of the fact that the latter does not glucosylate Rho. The receptors were blocked in HeLa cells by preincubation with an excess of TcdB_NAP1v_ at 4 °C to prevent toxin internalization. The cells were then incubated with either TcdB_NAP1_ or TcdB_VPI_ and switched to 37°C to initiate the intoxication process. At different time points, the successful entrance of the toxins was monitored by western blotting using an antibody that recognizes only unglucosylated RhoA. In non-blocked HeLa cells, RhoA was efficiently modified, with the same dynamics, by both TcdB_NAP1_ and TcdB_VPI_ (Figure 7A). On the other hand, in cells previously blocked with TcdB_NAP1V_, RhoA modification was only exerted by TcdB_VPI_, whereas no glucosylation was detected in TcdB_NAP1_-intoxicated cells for the duration of the experiment (Figure 7B). This experiment was repeated in 3T3 fibroblasts and Raw 264.7 macrophages with identical results (data not shown). To independently demonstrate that TcdB_NAP1_ and TcdB_VPI_ use different receptors, we took advantage of a recombinant fragment we used in previous works [26], encompassing residues 1349–1811 of TcdB_VPI_ and referred to as Receptor Binding Domain (RBD). HeLa cells were pre incubated with this fragment for 30 min at 4 °C to block toxin receptors. The cells were then treated with either TcdB_NAP1_ or TcdB_VPI_, and the percentage of cells displaying characteristic CPE were monitored. Under these conditions, RBD_VPI_ was able to confer a statistically significant protection from TcdB_VPI_ but not from TcdB_NAP1_ (Figure 7D), further supporting the notion that both toxins use independent cell entry mechanisms.

## 3. Discussion

Early studies from Stabler and colleagues showed that TcdBs from NAP1/027 strains exhibit significant sequence variation when compared to TcdBs produced by historical, non-epidemic strains (e.g., strain 630) [17]. Subsequent work in vitro [27], in the zebra fish model [19]**,** and in mouse intoxication assays [28] suggested that TcdB from *C. difficile* NAP1/027 strains is more cytotoxic than TcdB from historical strains. Thus, it was hypothesized that a ‘hypertoxic’ TcdB variant might contribute to the increased morbidity and mortality associated with ‘hypervirulent’ *C. difficile* NAP1/027 strains [28]. Lanis and colleagues suggested that the increased cytotoxic potential of TcdB_NAP1_ is explained by its ability to enter into target cells more rapidly and at an earlier stage in endocytosis [19] and by the fact that autoprocessing occurs more efficiently in TcdB_NAP1_ [29].

In the current study, we compared TcdB from the reference strain VPI 10463 (identical to TcdB from the 630 strain) with its TcdB counterparts from a NAP1 and a NAP1v strain [22]. In agreement with previous findings mentioned above, we also observed an increased biological activity of TcdB_NAP1_ in our mouse ligated intestinal loop model. However, our in vitro intoxication experiments with cultured cells revealed that TcdB_NAP1_ is not per se more cytotoxic than TcdB_VPI_. For instance, TcdB_VPI_ was capable of inducing cell rounding in 3T3 cells more efficiently than the TcdB_NAP1_ equivalent. This contrasts with previous findings from Stabler and colleagues, where it was shown that TcdB_NAP1_ is more cytotoxic than the reference TcdB in all tested cell lines, including 3T3 cells [27]. The authors used the 630 strain for native preparation of the reference TcdB, which produces an identical TcdB to that of the VPI 10463 strain. It is however of note that, in contrast to the VPI 10453 strain, the 630 strain does not overproduce toxin B [30]. In our hands, native toxin preparations from strain 630 yielded much less TcdB with decreasing activity due to ongoing degradation (data not shown). The latter fact might explain the differing cytotoxic activity of the NAP1 and 630 TcdBs observed by Stabler et al. in their cell intoxication experiments. From our data, we concluded that the difference in in vivo cytotoxicity of TcdB_NAP1_, TcdB_NAP1v_, and TcdB_VPI_ equivalents did not result from dissimilar in vitro cytotoxic activity of the toxins.

Accordingly, when we studied the autoprocessing of TcdB_VPI_, TcdB_NAP1_, and TcdB_NAP1v_ strains, we did not observe significantly relevant differences in the in vitro InsP6-induced autoprocessing efficiency between the toxins. Moreover, TcdB_VPI_ and TcdB_NAP1_ presented an identical substrate glucosylation profile and performed glucosylation with the same efficiency. We assume from these findings that the difference in in vivo biological activity of TcdB_NAP1_ and TcdB_VPI_ in mouse and zebra fish models might not be primarily based on a dissimilar efficacy and/or efficiency of their enzymatic subdomains.

Sequence comparisons between TcdBs from historical and hypervirulent strains revealed that the highest degree in sequence variation is found in the C-terminal part of the toxins [16,19,27]. Since the C-terminus of TcdB is involved in target cell interaction, Stabler and colleagues hypothesized already in 2008 that TcdB_NAP1_ may have a different binding capacity compared to less virulent TcdB counterparts [17]. To test this hypothesis, we took advantage of TcdB_NAP1v_ which, in contrast to TcdB_NAP1_ and TcdB_VPI_, does not modify RhoA in target cells. We preincubated HeLa cells with TcdB_NAP1v_ TcdB to block all cell surface receptors and additional host cell entry factors of the toxin, and, to our surprise, after intoxication with either TcdB_NAP1_ or TcdB_VPI_, RhoA modification still occurred in cells intoxicated with TcdB_VPI_. Thus, TcdB_NAP1v_ blocked host receptors and cell entry of TcdB_NAP1_ but not of TcdB_VPI_, indicating differential receptor specificity of the TcdB isoforms. This was further substantiated by the observation that the recombinant receptor binding domain of TcdB_VPI_ was able to protect the cells from TcdB_VPI_ but not from TcdB_NAP1_.

Cell surface interactions of TcdB are mediated by the combined repetitive oligopeptide (CROP) domain and an additional independent receptor-binding domain preceding the CROP domain (amino acid residues 1349 to 1811) [26,31]. Manse and Baldwin suggested the presence of even two additional receptor-binding domains in front of the CROP domain [32]. In recent years, three host receptors, namely, CSPG4, PVRL3, and FZDs, such as FZD2, were identified as TcdB receptors in the strain VPI 10463 [5,6,7]. Whereas CSPG4 is considered to interact with the CROP domain, PVLR3 and FZD2 bind to the additional receptor-binding domain(s) [6].

Very recently, the crystal structure of a TcdB fragment was solved in complex with the cysteine-rich domain of human FZD2 [25]. The TcdB fragment, referred to by the authors as FZD-binding domain (FBD), covers amino acid residues 1285–1804 and matches nearly exactly the delimitations of the additional receptor-binding domain (residues 1349–1811) discovered previously [26]. Recently, Chung et al. have shown that TcdB_NAP1_ exhibited only weak binding to FZD2 [33]. In line with this observation, the authors showed that a fragment of TcdB_VPI_ covering amino acid residues 1101–1836 (and which largely overlaps with the FBD described above) was capable of competitively inhibiting TcdB_VPI_ but not TcdB_NAP1_. Thus, it seems that TcdB_NAP1_ and TcdB_VPI_ mainly differ in the interaction with FZD2 due to variations in the FBD. Here, we compared the FBD sequences of TcdB_NAP1_ and TcdB_VPI_ and found that, among other residues, a phenylalanine at position 1597, crucially involved in the interaction of TcdB_VPI_ with FZD2, is not conserved in the TcdB_NAP1_ equivalent. The modification of FZD-interacting residues in TcdB_VPI_ resulted in a significant reduction of colonic TcdB-induced damage, establishing that FZDs are the main receptors that mediate TcdB_VPI_ intoxication [25]. However, even if this and other amino acids are not conserved in TcdB_NAP1_, the epithelial damage in the intestine produced by this toxin was severe, which suggests that different receptors mediate the uptake of TcdB_NAP1._

Within the C-terminus of TcdB_NAP1_, the so-called B2’ region (amino acid residues 1651–1852), represents the most variable sequence when compared to the reference TcdB (77% identity) [34,35]. Sequence variations in the B2’ region affect protein–protein interactions within TcdB and the exposure of neutralizing epitopes [34]. Intriguingly, a peptide spanning 19 amino acids in the B2’ region (amino acid residues 1769–1787, termed PepB2) was capable of inhibiting cytotoxicity caused by NAP1/027 and reference TcdB by disrupting cell binding due to the interaction with the CROP domain of the toxins. In contrast, the analogous peptide deriving from reference TcdB (PepB1) did not exhibit these inhibitory effects [36].

Taken together, our findings presented here support a model where TcdB_NAP1_ and TcdB_VPI_ utilize different or additional host receptors for cell entry, most likely as a result of sequence alterations in the FBD. The changed receptor specificity of TcdB_NAP1_ might have an influence on the cell tropism of the toxin, resulting in the increased virulence potential observed in vivo. Our data do not exclude that additional factors, such as the differences in the B2’ region mentioned above or sequence variations in the CROP domain, contribute to the increased in vivo cytotoxicity of TcdB_NAP1_.

## 4. Materials and Methods

### 4.1. C. difficile Strains and TcdB Sequence Analysis

In a previous study at the Laboratorio de Investigación de Bacteriología Anaerobia (LIBA) from the University of Costa Rica, strains NAP1 and NAP1v were isolated from stool samples following established protocols [22,37].

TcdB sequences from strains VPI 10493 (GenBank: MUJV01000001.1), 1470 (GenBank: Z23277.1), R20291 (NAP1, GenBank: FN545816.1), LIBA-5757 (NAP1v) (GenBank: NZ_LJCM00000000.1), and 8864 (GenBank: AJ011301.1) were extracted manually from WGS or directly from Genbank and aligned with MUSCLE using 100 iterations and default parameters. These alignments were transformed into dendrograms using PhyML. Thereafter, TcdB_VPI_ was selected as a reference, and discrepancies to it in the 1430–1600 region were highlighted with vertical black bars or colored residue letters using Geneious R11.

### 4.2. Native TcdB Purification and Recombinant Receptor Binding Domain Purification

TcdB was obtained from supernatants of strains grown in a dialysis system culture for 72 h in brain heart infusion broth (Difco, BD Life Sciences, Heidelberg, Germany) and purified as described previously [23]. The toxin was purified by anion-exchange chromatography followed by gel filtration (GE Healthcare, Chicago, IL, USA), and positive fractions were pooled and concentrated in Hepes buffer by ultrafiltration with a 100 kDa membrane. Proteins were quantified using the Bradford method (Bio-Rad, Hercules, CA, USA), and purity was assessed by SDS-PAGE and Coomassie staining. Final toxin identification was determined through mass spectrometry (LC–MS/MS), and only TcdB peptides were identified (not shown). Different toxin preparations of each TcdB were used for the various experiments and their replicates.

The RBD, comprising residues 1349–1811, from strain VPI 10463 was purified from *Bacillus megaterium* as previously described [26]. The C-terminal HIS-tagged fragment was purified by nickel affinity chromatography, and purity was assessed by SDS-PAGE and Coomassie staining.

### 4.3. Cultivation of Cells and Preparation of Cell Lysates

HeLa cells (ATCC CCL-2), 3T3 fibroblasts (ATCC CCL-163), and Chinese hamster ovarian (CHO) cells (kindly provided by Dr. Eugenia Corrales, University of Costa Rica, San José, Costa Rica) were cultured in DMEM supplemented with 10% FBS, 5 mM L-glutamine, penicillin (100 U/mL), and streptomycin (100 μg/mL). The cells were grown under humidified conditions with 5% CO_2_ and, unless indicated, they were incubated at 37 °C.

Cell lysates were prepared by washing the cells with PBS and lysing with precipitation buffer (1% Triton X-100, 0.1% SDS, 0.3% NonidetP-40, 500 mM NaCl, 10 mM MgCl_2,_ 50 mM Tris, pH 7.2). Lysates were centrifuged at 21,000× g for 10 min, and protein concentration was determined using the Bradford method (Bio-Rad, Hercules, CA, USA).

### 4.4. Murine Ileal Loop Model

Animal experimental procedures were approved by the University of Costa Rica Animal Care and Use Committee through CICUA 01-12 and CICUA 07-13 according to Law 7451: Bienestar de los animales, 26668-MICIT. Male Swiss mice, 20 to 25 g of weight, were subjected to fasting overnight and anesthetized with ketamine (60 mg/kg of body weight) and xylazine (5 mg/kg). Then, 10 µg of each toxin or Hepes control solution was injected into an ileal, loop following established protocols. The mice were sacrificed 4 h after inoculation, and the concentrations of the proinflammatory cytokines IL-1β and IL-6 in ileal tissue were determined [38]. The tissues were macerated in 1× PBS (pH 7.0), and cytokine concentration in the homogenates was determined by commercial ELISA, according to the instructions of the manufacturer (R&D Systems, Minneapolis, MN, USA). Intestinal sections were also fixed in formalin and stained with hematoxylin and eosin for histopathological evaluation according to previous protocols [38]. The samples were evaluated for the severity of epithelial damage and neutrophil infiltration using a histopathological score (HS) scale ranging from 0 (absence of alterations) to 3 (severe).

### 4.5. Cytotoxicity Assays

Confluent HeLa cells, 3T3 fibroblasts, and CHO cells grown in 96-well plates were intoxicated with 0.1 and 10 pM of TcdB from the different strains. The percentage of round cells in each well was evaluated every hour for a period of 12 h and then at 24 h.

For the cell death assay, confluent HeLa cells grown in 24-well plates were intoxicated with 100 pM of each toxin. Cytotoxicity was evaluated after 24 h of treatment. The cells were then harvested, washed in 1× PBS and resuspended in 100 µL 1× annexin binding buffer (Invitrogen, Waltham, MA, USA). Afterwards, 2 µL of Alexa Fluor 488 annexin V and 1 µL 100 mg/mL PI working solution (Invitrogen, Waltham, MA, USA) were added to the resuspended cells. After 15 min, the stained cells were analyzed by flow cytometry using a Guava easyCyte Mini (Merck Millipore, Burlington, MA, USA). The percentage of stained cells was determined with FLOWJO, LLC Data analysis software.

### 4.6. Rac1 and RhoA Glucosylation

Confluent HeLa cells grown in six-well plates were intoxicated with 100 pM of the corresponding toxins. Cell lysates were obtained at the indicated time points, and lysate proteins were separated by 10% SDS-PAGE and then transferred onto a PVDF membrane for western blotting. Rac1 and RhoA glucosylation were determined with monoclonal antibodies that do not recognize the modified isoforms, anti-Rac1 (clone 102; BD Transduction Laboratories, Franklin Lakes, NJ) and anti-RhoA (ab54835, Abcam, Cambridge, UK), respectively. For Rac1 glucosylation dynamics, β-actin was detected using rabbit anti-actin (A2066; Sigma-Aldrich, St. Louis, MO, USA).

### 4.7. In Vitro Glucosyltranferase Activity of TcdBs

A radioactive assay to determine the in vitro activity of each TcdB was performed as previously described [24]. For this, 10 µM of UDP-[14C]glucose, 10 µM of purified recombinant GST–GTPase, and 10 nM of each TcdB were mixed in glucosylation buffer (50mM HEPES, 100mM KCl; 2mM MgCl_2_, and 1mM MnCl_2_, pH 7.5). After 1 h of incubation at 37 °C, the reaction was stopped with loading buffer. Radiolabelled proteins were separated by 10% SDS-PAGE and visualized by autoradiography using a phosphorimager (GE Healthcare, Freiburg, Germany).

For the differential glucosylation assay, HeLa cells were treated with 10 pM of the corresponding TcdB for 12 h. Lysate proteins were used as substrates in a radioactive assay, following the conditions described above.

### 4.8. Ex vivo GTPase Activation Assay

The TcdB ability to inactivate Cdc42 was determined in HeLa cells grown in six-well plates. The cells were treated with 100 pM of each toxin for the indicated times. Cell lysates were centrifuged at 20,000× *g* for 1 min, and 20 μL were separated as a control for total amount of GTPase. The lysates were incubated with previously purified GST-tagged Rho binding domain coupled to glutathione-Sepharose beads. After 1 h of incubation at room temperature, the activated proteins were pulldown by centrifugation with the GST fusion effector protein. The proteins were separated by 10% SDS-PAGE and then transferred onto a PVDF membrane for western blotting. Cdc42 was detected using anti-Cdc42 antibodies (ab41429, Abcam, Cambridge, UK).

### 4.9. In Vitro Cleavage Assay

The in vitro autoprocessing activity of TcdBs was performed in a 20 uL reaction with 200 nM of toxin, cleavage buffer (150 mM NaCl, 20 mM Tris pH 7.4), and 10 mM InsP6 (Sigma-Aldrich, St. Louis, MO, USA) [23]. The reaction was incubated for 0, 2.5, 5, 7.5, 10, 20, 30, 60, and 90 min at 37 °C. The reaction was stopped by addition of loading buffer and boiling at 95 °C for 5 min. The samples were separated by 7.5% SDS-PAGE and then analyzed by Coomassie staining. The activity of each toxin was quantified by comparing the densities of the bands of full-length and processed protein using Image J software (NIH, Bethesda, MD, USA). The data were plotted using nonlinear regression and in GraphPad Prism software (GraphPad Software, Inc., San Diego, CA, USA [29].

### 4.10. Uptake Competition Assay

Prior to blockade and intoxication, confluent HeLa cells were incubated at 4 °C for 30 min. For NAP1v blockade, 1 nM of TcdB_NAP1v_ was added to the precooled cells. After incubation for 30 min at 4 °C, the cells were treated with 10 pM of TcdB_VPI_ or TcdB_NAP1_ and incubated at 37 °C. Cell lysates were obtained at the indicated time points, and lysate proteins were separated by 10% SDS-PAGE and then transferred onto a PVDF membrane for western blotting. RhoA glucosylation by TcdB_VPI_ and TcdB_NAP1_ was determined with a monoclonal antibody that does not recognize the modified isoform, anti-RhoA (ab54835, Abcam, Cambridge, UK); β-actin was detected using rabbit anti-actin (A2066; Sigma-Aldrich, St. Louis, MO, USA). For the receptor binding domain blockade, 100 molar excess of recombinant toxin fragment was added to the cells. After incubation for 30 min at 4 °C, the cells were treated with 100 pM of TcdB_VPI_ or TcdB_NAP1_ and incubated at 37 °C. The percentage of round cells in each well was evaluated after 60 min.
